# Non-Tuberculous Mycobacteria and the Performance of Interferon Gamma Release Assays in Denmark

**DOI:** 10.1371/journal.pone.0093986

**Published:** 2014-04-04

**Authors:** Thomas Stig Hermansen, Vibeke Østergaard Thomsen, Troels Lillebaek, Pernille Ravn

**Affiliations:** 1 Department of Thoracic Medicine and Infectious Disease, Nordsjaelland Hospital, Hillerød, Denmark; 2 International Reference Laboratory of Mycobacteriology, Statens Serum Institut, Copenhagen, Denmark; Hopital Raymond Poincare - Universite Versailles St. Quentin, France

## Abstract

**Background:**

The QuantiFERON-TB-Gold Test (QFT) is more specific than the Mantoux skin-test to discriminate between *Mycobacterium tuberculosis* (MTB) and non-tuberculous mycobacterial (NTM) infections. Here we study the performance of the QFT in patients with NTM disease.

**Methods:**

From 2005 to 2011, nationwide patient data on positive NTM cultures (n = 925) were combined with nationwide data on QFT results (n = 16,133), both retrieved from the International Reference Laboratory of Mycobacteriology, Denmark. A total of 112 patients with NTM infections had a QFT performed, 53 patients had definite NTM disease, 10 had possible disease and 49 had NTM colonization.

**Results:**

QFT was positive in 8% (4/53) of patients with definite disease, 40% (4/10) with possible disease and 31% (15/49) with colonization. Positivity rate was lowest among patients with definite disease infected with NTM without the RD1 region 4% (2/50). None of the 15 children with MAC lymphadenitis had a positive QFT.

**Conclusion:**

This study is one of the largest assessing IGRAs in patients with NTM disease in a TB low-incidence setting. Our study showed that the QFT holds potential to discriminate between NTM and MTB infections. We found no positive IGRA test results among children with NTM not sharing the RD1-region of MTB resulting in a 100% specificity and we suggest that a QFT in a child presenting with cervical lymphadenitis may be helpful in distinguishing NTM from TB lymphadenitis.

## Introduction

Non-tuberculous mycobacteria (NTM) are a ubiquitous group of mycobacteria found in the environment worldwide (soil, dust, animals, and tap water) [1], [2]. They are opportunistic pathogens causing human disease, especially in immuno-compromised individuals. Studies report increasing numbers of both NTM disease and NTM colonization [3]–[8], and the prevalence varies according to geographic region. The clinical impact of NTM infections is difficult to assess due to difficulties in discriminating between disease and colonization. To aid in the diagnosis and treatment of pulmonary NTM disease, the American Thoracic Society (ATS) and the Infectious Disease Society of America (IDSA) have issued a set of guidelines regarding pulmonary NTM disease that is useful in discriminating disease from colonization [9]. These guidelines are based on a combination of clinical, radiographic, and microbiologic criteria that must be met to make a diagnosis of NTM lung disease. However, in the absence of clinical and radiographic information, C. Andrejak et al have successfully classified NTM lung disease according to modified (stricter) ATS microbiologic criteria [10]. Extra pulmonary NTM disease is easier to diagnose, because NTM cultured from outside the lungs has been suggested to represent clinically significant disease in almost all cases [11].

Differentiation between NTM infection and tuberculosis (TB) can be difficult. For more than a century, the Tuberculin skin test (TST or Mantoux) has been used for detecting latent tuberculosis infection (LTBI) and sometimes as an additional diagnostic tool for active TB. The TST suffers from suboptimal sensitivity and it is unreliable in distinguishing *Mycobacterium tuberculosis* (MTB) infection from infection with NTM and previous BCG-vaccination [12].

Immunodiagnostic tests such as the Interferon-gamma (IFN-γ) release assays (IGRAs) are more specific and are based on the T-cell mediated IFN-γ release after stimulation with specific MTB antigens. These tests have shown better specificity worldwide with no cross reactivity with most NTM [13] and no BCG cross reactivity, and they have a higher sensitivity compared to the TST for the diagnosis of both active TB and LTBI [14], [15], especially in immunosuppressed individuals [16]–[22]. The QuantiFERON-TB-Gold-Test (QFT) is based on response to the MTB specific peptide antigens ESAT-6, CFP-10 and TB7.7 which are located in a specific genomic area in MTB, called the region of difference (RD1). The RD1 is present in mycobacteria belonging to the *M. tuberculosis* complex (*M.tuberculosis, M. africanum, M. bovis, M. canettii, M. caprae, M. microti, M. pinnipedii, M. mungi and M. orygis*) [23] and very few NTM species also share the RD1 of MTB (*M. gastri, M. kansasii, M. marinum, M. riyadhense* and *M. szulgai*) [13], [24], [25]. Infection with these strains can potentially result in a positive QFT-result [13]. In the vaccine strain *M. bovis* BCG and in the majority of NTMs, the RD1 is absent. Thus, in BCG-vaccinated and the vast majority of individuals infected with NTM, the QFT should be negative. Due to the high specificity of the QFT, some studies have suggested that it can be used to discriminate between infection with MTB and NTM [26]-[29].

The aim of this study is to assess the performance of the QFT in patients with NTM disease.

## Material and Methods

### Ethics statement

The project was approved by the Danish Data Protection Agency (Jr. nr. 2011-54-1230). Written consent was not obtained from the participants because according to the regional health research ethics committee approval, the study is not a “clinical trial” but a “retrospective survey” based on routine register data analyzed anonymously. The regional health research ethics committee (De Videnskabsetiske Komiteer, Region Hovedstaden Kongens Vænge 2, 3400 Hillerød, Denmark) evaluated the project and found it to be “not notifiable” and waived the need for written informed consent. The enquiry to the ethical committee can be documented (protocol number: H-1-2012-FSP 90).

### Design

The study is a retrospective register based study comparing QFT results in patients with culture verified NTM disease or colonization from January 2005 to February 2011 based on nationwide data from the International Reference Laboratory of Mycobacteriology at Statens Serum Institut.

### Study population

Analyses of the QFT performance in patients with positive NTM culture were performed on patients who had ≥1 QFT performed and positive NTM culture(s). We did not have access to complete data on clinical- and radiographic features and instead we used modified ATS/IDSA 2007 criteria based solely on microbiologic data as described in [10]. This approach has been used and validated by Andrejak et al in 2010 [10] to classify patients into three categories on the basis of microbiological data: definite NTM disease, possible NTM disease, and NTM colonization. In addition, we included patients with NTM cultured from extra pulmonary sites as cases with definite disease in line with Freeman et al [11].

Patients were excluded if their QFT was performed ≥6 month before and/or ≥1 month after the positive NTM culture, they had discordant QFT results, they had a history of culture verified TB, or if their NTM species were not identifiable.

### Laboratory analyses

#### Mycobacterial cultures

After NaOH-NALC pretreatment of non-sterile specimens, the sediment from the specimens was cultured on Löwenstein–Jensen slants at 35°C and MGIT at 37°C for a maximum of 56 days. Species identification was carried out by either InnoLipa species (InnoGenetics, Ghent, Belgium) and/or 16S DNA sequencing using primers directed at hypervariable region A.

#### QFT analyses

The QFT was used (Cellestis, Ltd, Carnegie, VIC, Australia). After initial incubation and centrifugation, plasma was stored at 2–8°C until analysis. ELISA was performed in accordance with manufacturer's instructions. For the QFT assay heparinized whole blood were incubated for 16–24 h in precoated tubes within less than 16 h from sampling. The QFT tubes were pre-coated with a mixture of synthetic MTB specific peptides representing ESAT-6, CFP-10 and TB7.7 as test antigens, phytohaemagglutinin (PHA) (positive (mitogen) control) or saline (negative (nil) control). The concentration of IFN-γ in the plasma was determined using the recommended ELISA test kit. QFT results were calculated using the software provided by the manufacturer.

### Literature search on IGRA performance in patients with NTM

In order to collect all published information on the performance of IGRA in patients with NTM disease, a PubMed search of the literature was done to identify articles on NTM and IGRA test performance. The following search terms were used: “IGRA”, “Non tuberculous mycobacteria”, “QuantiFERON”, “NTM”, “atypical mycobacteria”, “IGRA and NTM” and “predictive value and IGRA” and only papers in English Language were included. The references of retrieved articles were also reviewed to identify additional sources. No date restrictions were placed on these searches. Studies eligible for inclusion were studies in humans where patients had an IGRA test performed and culture verified NTM disease.

### Statistical analysis

Pearson's chi-square test was used in evaluation of categorical variables. Mann-Whitney U was used to evaluate continuous variables. Baseline patient characteristics are expressed as median and interquartile range (continuous variables) and as numbers and percentages (categorical variables).

A two-sided P-value<0.05 was considered significant.

## Results

### QFT performance and non tuberculous mycobacteria

**Figure 1:**
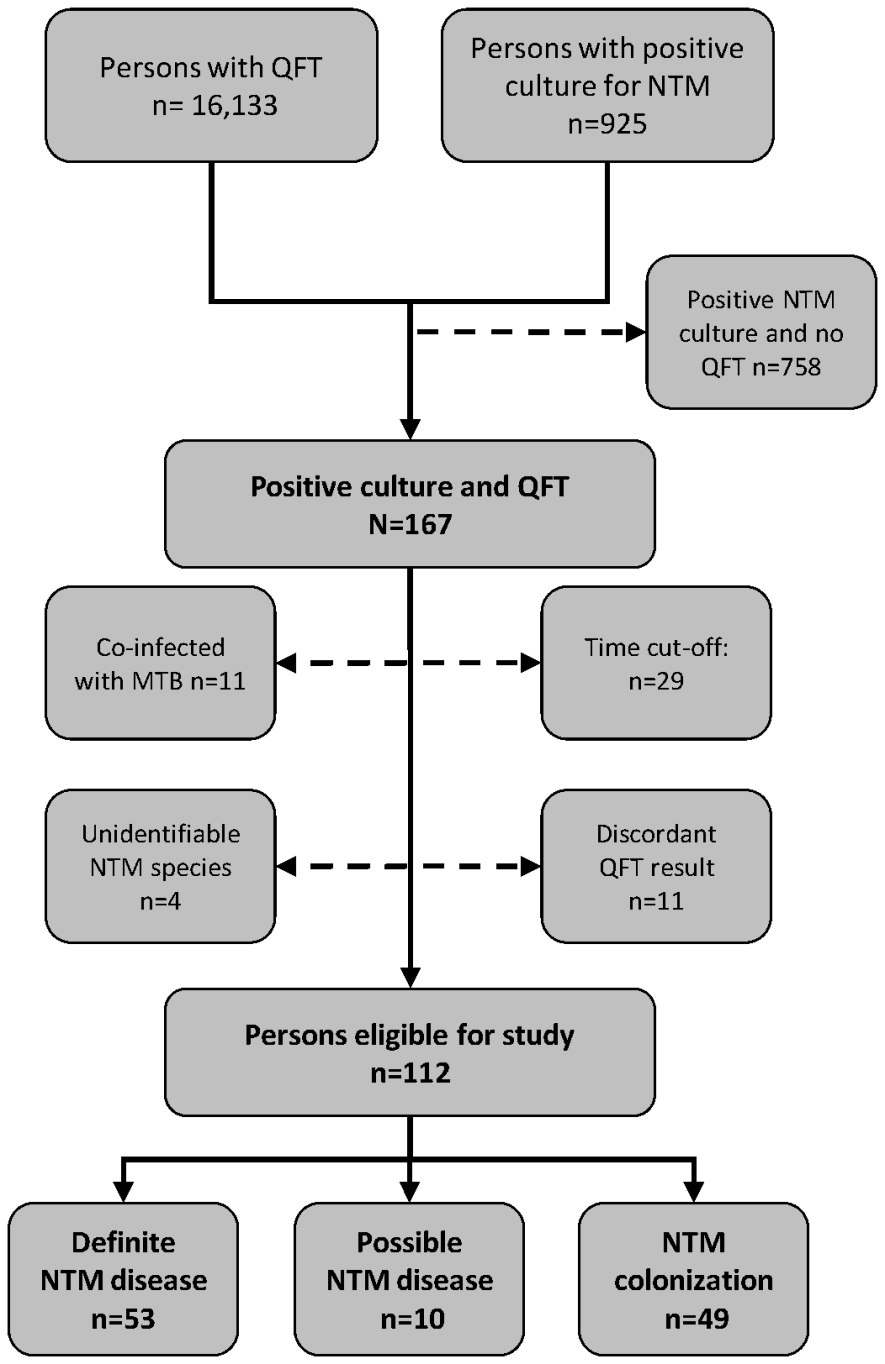
Flowchart study population. Figure 1 illustrates the inclusion and exclusion of persons with positive NTM culture and the classification of patients into three categories on the basis of microbiological data: definite NTM disease, possible NTM disease and NTM colonization.

In total 925 patients had a positive NTM culture, 16,133 persons had a QFT performed and 167 persons had both QFT and NTM results available (Figure 1). Of these 55 persons were excluded; 29 because they had the QFT performed ≥6 month before or ≥1 month after NTM culture(s) were performed, 11 had discordant QFT results, 11 were infected with both MTB and NTM, and in 4 cases the mycobacteria could not be identified to the species level. One person with a QFT had both *M. gordonae* and *M. abscessus-chelonae* detected, and was classified as *M. abscessus-chelonae* as this is the most pathogenic species of the two. Thus, 112 persons were available for evaluation of the QFT performance. Table 1 shows baseline characteristics of the 112 persons with a positive NTM culture and a QFT in comparison with persons who had a positive NTM culture (n = 758) but no available QFT. We found a significant difference between the groups in terms of age (P = 0.036). Also, among the persons with a QFT, a larger proportion (34%) had *M*. *gordonae* isolated compared to the group without QFT (16%) (p<0.001, Table 1).

**Table 1:** Baseline characteristics of persons with a positive NTM culture.

	NTM total (n = 925)	NTM and QFT(n = 112¤)	NTM, no QFT (n = 758)	p value*
**median age (IQR)**	60 (37)	55 (33,5)	62 (38,7)	P = 0.036**
**sex (male %)**	51	56	50	P>0.1***
**NTM species n(%)**				
*M. abscessus-chelonae*	44 (5)	4 (4)	40 (5)	P>0.1
*MAC*	460 (50)	48 (43)	392 (52)	P>0.1
*M. celatum*	28 (3)	5 (4)	23 (3)	P>0.1
*M. fortuitum-peregrinum*	24 (3)	2 (2)	22 (3)	P>0.1
*M. gordonae*	169 (18)	38 (34)	118 (16)	P<0.001
*M. kansasii*	18 (2)	2 (2)	15 (2)	P>0.5
*M. malmoense*	42 (5)	6 (5)	33 (4)	P>0.1
*M. marinum*	21 (2)	1 (1)	18 (2)	P>0.1
*M. xenopi*	41 (4)	3 (3)	34 (4)	P>0.1
Other species	78 (7)****	3 (3)	63 (7)	P>0.1

¤ n = 55 persons excluded, see Figure 1.

*Comparison between “NTM and QFT” and “NTM, no QFT”.

**Mann-Withney U test.

***Chi squared.

****In this category persons with more than 1 NTM species isolated are included (n = 8).

Total percentages add up to 99–101 because of rounding.

Table 1 shows the distribution of NTMs isolated in the different groups of participants with or without a matching QFT.

The 112 persons included in the final analysis were divided according to clinical significance into 3 diagnostic groups [10], [11]; definite disease (n = 53), possible disease (n = 10) and colonization (n = 49). Among those with definite disease we found 8% positive and 9% indeterminate QFT results. Similarly among patients with possible disease we found 40% positive and 10% indeterminate and among those with colonization 31% positive and 6% indeterminate (Table 2).

**Table 2:** QFT-performance in persons with positive NTM culture (n = 112).

	QFT-test Positive n(%)	QFT test Indeterminate n(%)	QFT-test Negative n(%)	Total
**Patients with NTM disease n (%)** *	4 (8)	5 (9)	44 (83)	53 (100)
**Patients with possible NTM disease n (%)** **	4 (40)	1 (10)	5 (50)	10 (100)
**Persons with NTM colonization n (%)** ***	15 (31)	3 (6)	31 (63)	49 (100)

*Table 3.

**5 with MAC, 3 with *M. gordonae* and 2 with *M. xenopi*.

***2 with *M. abscessus-chelonae*, 9 with MAC, 2 with *M. fortuitum*, 35 with *M*. *gordonae* and 1 with *M. malmoense*.

Table 2 shows the QFT results in patients with positive NTM culture divided into 3 groups according to clinical significance.

Among 53 patients with definite NTM disease, 50 (94%) were infected with NTM species not known to share RD1 and their QFT-result were: 2 (4%) positive, 43 (86%) negative, and 5 (10%) indeterminate (Table 3a). Three patients had definite disease due to an NTM species sharing the RD1 of whom 2 patients with *M*. *kansasii* had a positive, and 1 patient with *M. marinum* had a negative QFT result (Table 3b).

**Table 3:** QFT-performance in patients with disease due to non tuberculous mycobacteria according to RD1 (n = 53).

Table 3a: Patients with NTM not sharing RD1	QFT-test Positive n(%)	QFT test Indeterminate n(%)	QFT-test Negative n(%)	Total
*M. abscessus-chelonae*	1 (50)	0	1 (50)	2
*MAC*	0	5 (15)	29 (85)	34
*M. celatum*	1 (20)	0	4 (80)	5
*M. interjectum*	0	0	1 (100)	1
*M. malmoense*	0	0	5 (100)	5
*M. shimoidei*	0	0	1 (100)	1
*M. simiae*	0	0	1 (100)	1
*M. xenopi*	0	0	1 (100)	1
**TOTAL**	2 (4)	5 (10)	43 (86)	50

Table 3b: Patients with NTM sharing RD1	QFT-test Positive n(%)	QFT test Indeterminate n(%)	QFT-test Negative n(%)	Total
*M. kansasii*	2 (100)	0	0	2
*M. marinum*	0	0	1 (100)	1
**TOTAL**	2 (67)	0	1 (33)	3

Tables 3a and 3b show the results of the QFT in patients with definite NTM disease. The patients are divided in 2 groups according to the likely cross reaction with the RD1 antigenic region of *M. tuberculosis*.

Definite disease was most frequently due to MAC and constituted 34/53 (64%) of the patients. Five (15%) had an indeterminate QFT result and none had a positive result. In 17 children aged 0–9 years MAC was identified in 15 cases and all had a valid QFT (not shown). In the children with MAC we found no positive QFT results and MAC was solely found in biopsies from extra pulmonary lymph nodes. In contrast, among 19 adult patients with MAC disease 17/19 (89%) of the samples were pulmonary and the last two had disseminated disease (blood and faeces). Interestingly 5/19 (26%) in this group could not produce a valid test (data not shown).

### Review of IGRA results among patients with NTM disease

In order to evaluate the performance of IGRA in patients with NTM disease, we compared our result with comparable published studies (Table 4). We identified 9 studies assessing IGRA tests in patients with pulmonary NTM disease defined by the ATS/IDSA criteria [26], [28], [30]–[36] and 2 studies assessing IGRA tests in patients with extra pulmonary NTM disease [29], [37]. Overall we found 478 patients ranging from 1–214 participants in the individual studies. The studies were of very heterogeneous design and geographical origin and were not directly comparable in regard to TB exposure or history: some studies used active TB or known exposure as an exclusion criteria [26], [34], whereas others did not [30], [32], [33]. After excluding 16 cases that had indeterminate results we found the overall rate of positive QFT to be 18% (81/462). Among patients infected with NTM not sharing the RD1, we found a pooled positivity rate of 12% (48/404) ranging from 0–50% in the different studies. In the group of patients infected with NTM known to share RD1 (*M*. *kansasii, M*. *marinum* and *M. szulgai*), 57% (33/58) had a positive QFT ranging from 52–100%. In the available literature as well as in our study MAC was the most common NTM found and among 371 patients with MAC disease the pooled positivity rate was 10% (37/371). The largest single study included 214 patients with no risk factors for TB, and found a positivity rate for MAC of only 1% (2/163) [26], this is comparable to our study where we find 0/34 positive QFT among patients with MAC disease.

**Table 4:** Overview of the published studies on IGRA response among patients with non tuberculous mycobacterial disease.

Ref #	N =	Age	Sex	Country	TB incidence	Type of IGRA	Disease	Species found	IGRA positivity rate	IGRA positivity rate
		mean	M %				localization	(%)	NTM with RD1	NTM with no RD1
									n/n (%)	n/n (%)
[37]	1	66	100	Switzerland	7,6	QFT-GIT	Uveitis	*M. kansasii (100)*	**1/1** (100)	
[31]	8	68,5	38	Japan	21	QFT-GIT	Lung	*MAC (100)*		**4/8** (50)
								*M. abscessus(12,5)*#		
[32] *	40	61	48	Korea	71,6	QFT-G	Lung	*MAC (100)*		**12/35** (34)
[33]+	59	59,6	51	Korea	71,6	QFT-GIT	Lung	*MAC (61)*		**10/36** (36)
								*M. abscessus(30)*		**7/18** (39)
								*M fortuitum (7)*		**3/4** (75)
								*M. szulgai (2)*	**1/1** (100)	
[26] ##	214	62,4	30	Japan	22	QFT-G	Lung	*MAC* 163 (76)		**2/163** (1)
								*M. kansasii (15)*	**17/33** (52)	
								*M. marinum (6)*	**7/12** (58)	
								*M. szulgai (1)*	**1/3** (33)	
								*M. abscessus (1)*		**0/3** (0)
[34] **	14	65,8	21	USA	4,2	T-SPOT	Lung	*MAC (100)*		**1/14** (7)
[35]	3	65	67	Netherland	5,9	T-SPOT	Lung	*MAC (25)*		**0/3** (0)
								*M.genavense(25)*		
								*M.malmoense (25)*		
[29]++	23	3,7	30	Germany	6	QFT-GIT	Lymph node	*MAC (90)*		**0/19**
								*M. scrofolaceum (5)*		
								*M. simiae (5)*		
[30] ###	8	N.A.	N.A.	Taiwan, rep.	68	T-SPOT	Lung	*MAC (25)*		**1/2** (50)
				China				*M.abscessus/chelonae (50)*		**1/4** (25)
								*M. marinum (12,5)*	**1/1** (100)	
								*M. kansasii (12,5)*	**0/1** (0)	
[36] ***	100	64,4	38	Japan	23	QFT-G	Lung	*MAC (94)*		**7/87** (8)
								*M. kansasii (4)*	**4/4** (100)	
								*M. marinum (2)*	**2/2** (100)	
[28]	8	63	13	USA	5,5	ESAT-6	Lung	*MAC (100)*		**0/8** (0)
**[Present**	53	55	56	Denmark	7	QFT-GIT	Lung/	*MAC (64)*		**0/34** (0)
**study]+++**							Extra pulm.	*M. malmoense (9)*		**0/5** (0)
								*M. celatum (9)*		**1/5** (20)
								*others (11)*		**1/6** (17)
								*M. kansasii/marinum (6)*	**2/3** (67)	
**Total**									**34/58** (59)	**48/404** (12)

# 1 co-infected with MAC and *M. abscessus*.

*5 patients are excluded due to indeterminate results, 17.5% had a previous history of TB

+31% had a previous history of TB.

## Patients with a history of TB were excluded

**Patients with a history of TB and TB contacts excluded.

++4 Patients were excluded due to indeterminate results.

### Study of patients suspected of TB.

***7 Patients were excluded due to indeterminate results.

+++Data on these patients are not included in the “Total” column below. Age is expressed as median. “Others” constitute *M. abscessus/chelonae, interjectum, shimoidei,simiae and xenop*i (see Table 3a).

Abbreviations: N.A.: not available.

QTF-2G: QuantiFERON TB 2 Gold, Cellestis.

QTF-GIT: QuantiFERON TB Gold-In-Tube, Cellestis.

IGRA: Interferon-gamma release assay.

MAC: Mycobacterium avium-intracellulare complex.

T-SPOT: T-SPOT.TB, Oxford Immunotec.

## Discussion

This is one of the largest studies assessing IGRA performance in patients with NTM disease in a TB low burden population. Among 53 patients with NTM disease MAC predominated. Adults presented with pulmonary MAC disease and we confirmed the well-known association of children presenting with MAC lymphadenitis [9]. We found an overall positivity rate of 8%. The rate was lower among patients with NTM without the RD1 region (4%), and interestingly, no patients with MAC including 15 children with MAC lymphadenitis were QFT positive. Our study showed that in Denmark, a low TB incidence country, the QFT holds potential to discriminate between NTM and MTB infections, suggesting that a negative QFT in a child presenting with cervical lymphadenitis may be helpful in distinguishing NTM from TB lymphadenitis.

Interestingly the rate of positive QFT results was much lower in the group with definite disease compared to the groups with possible NTM disease and NTM colonization. An explanation for this finding could be that a large proportion of the NTMs isolated from possible NTM disease and NTM colonization was found in gastric aspiration which was mainly performed in patients highly suspect of active TB due to clinical symptoms, ethnicity or known exposure and at risk of LTBI.

We assessed the performance of IGRA in patients with NTM disease and compared our results with available data: The positivity rate of the QFT in our study was 8% which is lower than the positivity rate of 18% we found when pooling the data from available studies (Table 4). Further, we showed that the QFT positivity rates varied depending on the presence or absence of RD1 in the NTM; in the NTM group with no RD1, low positive rates were found at 4%, which was lower than the pooled rate of 12% from available publications on the subject. The available studies comprised high TB-incidence populations rendering LTBI more likely compared to our TB low incidence population. Two out of three patients with *M. marinum* and *M. kansasii* isolates had a positive QFT in line with the literature study showing a pooled positivity rate of 57% (33/58) among the group with presence of the RD1 region. In patients with *M. marinum* and *M. kansasii*, QFT cannot be used to discriminate between NTM and MTB as the IGRA is expected to be positive in both groups. In summary, although IGRA could provide some information to the clinician there is a risk of “false positive IGRA” in a TB high-incidence population, with a large proportion of LTBI and a high a priori risk of TB.

Clinically, there is a need for rapid means to discriminate lymph node TB from NTM lymphadenitis. Griffith et al. states [9] that if a child with granulomatous disease has a strongly positive TST, anti-TB therapy is recommended until the result of lymph node culture is available, likely resulting in overtreatment. All children with MAC lymphadenitis in our study had a negative QFT and there were no indeterminate results indicating a high (100%) specificity and negative predictive value. In an on-going evaluation of the QFT performance in patients with active TB in Denmark, data from 16 children aged 1–14 years with confirmed TB revealed a sensitivity of 100% (16/16) (data not shown). Together with the study by Detjen et al [29], our data indicate high sensitivity and specificity of the QFT in discriminating between TB and MAC in children. Thus, we suggest that a negative QFT in a child presenting with cervical lymphadenitis may be very helpful in ruling out TB in low endemic regions.

Our study holds some limitations; Information on radiographic and clinical diagnostic criteria were not available, but we used modified microbiological criteria for NTM pulmonary disease and we considered samples of extra pulmonary origin to be clinically significant according to cases [11]. The direct comparisons on IGRA performance among patients with NTM disease was difficult as the studies varied with respect to incidence of NTM disease, study design, sample sites, diagnostic criteria and geographical distribution [9], [38]–[40].

## Conclusion

This study is one of the largest assessing IGRA performance in patients with NTM disease in a low TB-incidence setting. We showed that QFT holds potential to discriminate between NTM and MTB infections. We found an overall low positivity rate in patients with NTM without RD1 (4%), especially in children with MAC lymphadenitis (100% QFT negative). Thus, in a child presenting with cervical lymphadenitis a negative QFT may be helpful in distinguishing NTM from TB lymphadenitis. This finding needs to be studied prospectively in a larger population.
